# Review on the Antimicrobial Properties of Carbon Nanostructures

**DOI:** 10.3390/ma10091066

**Published:** 2017-09-11

**Authors:** Ahmed Al-Jumaili, Surjith Alancherry, Kateryna Bazaka, Mohan V. Jacob

**Affiliations:** 1 Electronics Materials Lab, College of Science and Engineering, James Cook University, Townsville, QLD 4811, Australia; Ahmed.Aljumaili@my.jcu.edu.au (A.A.-J.); Surjith.Alancherry@my.jcu.edu.au (S.A.); kateryna.bazaka@qut.edu.au (K.B.); 2 School of Chemistry, Physics, Mechanical Engineering, Queensland University of Technology, Brisbane, QLD 4000, Australia

**Keywords:** carbon nanostructures, antimicrobial properties, fullerene, carbon nanotubes, graphene, diamond-like carbon

## Abstract

Swift developments in nanotechnology have prominently encouraged innovative discoveries across many fields. Carbon-based nanomaterials have emerged as promising platforms for a broad range of applications due to their unique mechanical, electronic, and biological properties. Carbon nanostructures (CNSs) such as fullerene, carbon nanotubes (CNTs), graphene and diamond-like carbon (DLC) have been demonstrated to have potent broad-spectrum antibacterial activities toward pathogens. In order to ensure the safe and effective integration of these structures as antibacterial agents into biomaterials, the specific mechanisms that govern the antibacterial activity of CNSs need to be understood, yet it is challenging to decouple individual and synergistic contributions of physical, chemical and electrical effects of CNSs on cells. In this article, recent progress in this area is reviewed, with a focus on the interaction between different families of carbon nanostructures and microorganisms to evaluate their bactericidal performance.

## 1. Introduction

**Figure 1:**
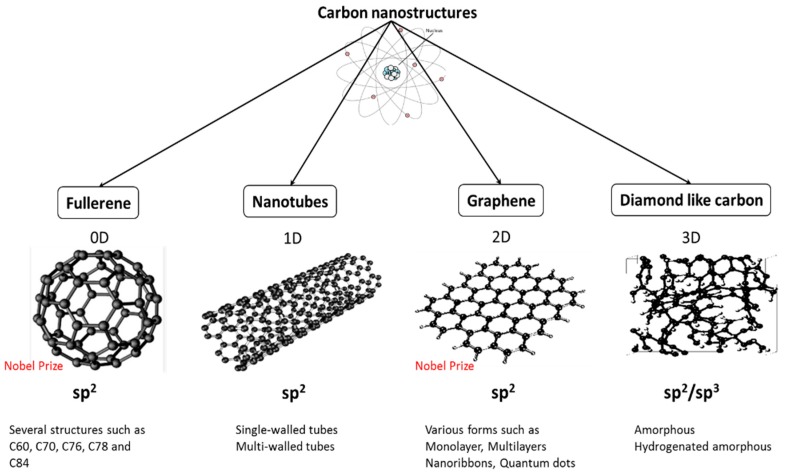
Several forms of carbon nanostructures.

Nanotechnology is a swiftly rising field that significantly contributes to the present progress in the development of effective biomaterials. In order to keep the growth of advancement at the present pace, innovative nanomaterials with novel and unique properties are required. Among various nanomaterials, carbon nanostructures (CNSs) and their derivatives gained significant attention due to their extraordinary properties and potential to apply them in a vast number of existing and emerging applications [1,2,3,4]. Indeed, carbon can bond to itself in a unique architecture to form extremely low-dimension structures, including fullerenes (0D), nanotubes (1D), graphene sheets (2D) and diamond-like carbon (3D), as seen in Figure 1. In general, CNSs are recognized for their excellent electrical conductivity, supreme mechanical strength, high thermal conductivity, extraordinarily high surface area, excellent photoluminescent properties [5], high transparency and structural stability [6,7]. These unique properties make carbon nanoarchitectures promising for applications stretching from thin film transistors [8], transparent conducting electrodes [9], photovoltaics [10], supercapacitors [11], to biosensors [12], drug delivery [13], tissue engineering [14] and photothermal therapy [15]. 

CNSs offer promising potential to engage with biological molecules [16]. In particular, a number of carbon-based nanomaterials have been found to possess powerful bactericidal properties toward pathogenic microorganisms. The mechanism by which CNSs inactivate bacteria is complex and depends on intrinsic properties of CNSs, e.g., composition and surface modification, the nature of the target microorganisms, and the characteristics of the environment in which cell-CNS interactions take place [17]. 

In principle, the bactericidal action of CNSs typically involves a combination of physical and chemical mechanisms [17,18,19]. Physically, CNSs may cause considerable structural damage to the cell wall and membrane of the microorganism. Furthermore, carbon nanomaterials such as graphene sheets are capable to biologically isolate cells from their microenvironments, which may eventually lead to cell death [20]. Chemical interaction between CNSs and the microorganism surface may lead to generation of toxic substances, such as reactive oxygen species (ROS), placing the cell under oxidative stress. The interactions between CNSs and cells may cause an electron transfer phenomenon, where electrons are progressively drained from the microbial outer surface, which may cause ROS-independent oxidative stress, leading to the biological death [21].

This manuscript reports the key advancements in the use of several carbon nanostructures including fullerene, carbon nanotubes (CNTs), graphene and diamond-like carbon (DLC) as antibacterial agents. Also, it will critically focus on the antibacterial mechanisms/performance of CNSs and highlight the influence of various factors (e.g., size, light, modifications) on their toxicological profile toward microorganisms.

## 2. Antimicrobial Performance of Carbon Nanostructures

CNSs are being successfully employed in biological-related studies such as sensing, biomaterials, drug delivery, and antibacterial agents [22]. There is a vast body of relevant monographs and reviews in literature discussing the biological/bactericidal activities of carbon nanostructures (see e.g., [17,23,24,25,26,27,28]), with many more relevant publications emerging rapidly. Examples used in this review have been selected only to highlight particular favourable or limiting aspects in the property, characterization, and application of carbon nanostructures. Using this information and select references as a platform, we strongly encourage the reader to further explore this rapidly growing and highly-promising research field. In this section, we focus on the antimicrobial performance/mechanism of several carbon allotropes. Table 1 summarizes the antimicrobial properties of several forms of CNSs.

## 3. Fullerene

There are many published reports that demonstrate biological activity of fullerene-caged particles [31,43,44]. Several mechanisms have been proposed to describe the bactericidal action of fullerene materials. 

**Figure 2:**
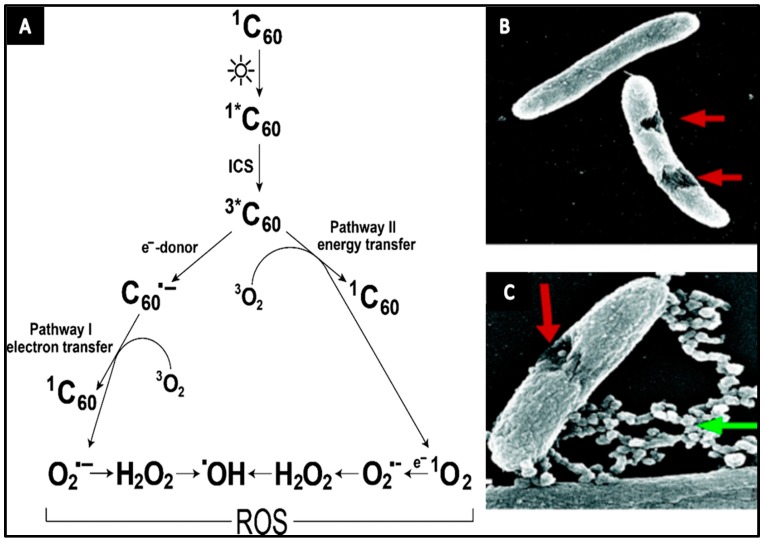
(**A**) Schematic representation of fullerene C_60_ photochemical pathways leading to reactive oxygen species (ROS) generation. Reprinted with permission from Reference [46]; (**B**,**C**) SEM images of *S. oneidensis* MR-1 cells treated with C_60_−NH_2_ show cellular damage. Cell samples were fixed for SEM images approximately 1 h after exposure to 20 mg/L C_60_−NH_2_. Green arrow points to nanoparticle aggregations and red arrow points to the damaged part of the cell. Reprinted with permission from Reference [56].

In particular, fullerenes and their derivatives have demonstrated powerful antibacterial activity against a wide spectrum of microorganisms when exposed to light [29,30]. One possible explanation for this bactericidal behavior is related to the unique structure of the fullerene particle. From structural design point of view, fullerene is a closed-cage nanoparticle, where the conjugation is extended through π-electrons. This structure is perhaps the main reason that fullerenes can absorb light and subsequently generate reactive oxygen species [27]. As soon as fullerene (C_60_) is illuminated by photons, C_60_ will excite from the ground state to an extremely short-lived (~1.3 ns) excited state. The excited state quickly decays to a lower triplet state that has a longer lifetime (50–100 μs) [45]. Then, in presence of molecular oxygen (^3^O_2_), fullerene may produce ROS, including singlet oxygen (^1^O_2_), through energy transfer photochemical pathway and superoxide anion (O_2_^−^) through electron transfer pathway, as illustrated in Figure 2A [46]. These radicals are short-lived oxidants containing one or more unpaired electrons excited in their highest occupied atomic/molecular level [47,48]. ROS are generally accepted to be responsible for eukaryotic lipid peroxidation and eukaryotic cell membrane interruption [29,49,50,51]. High level of ROS is acutely lethal to microorganisms [52], triggering damage to cellular molecules like lipids, proteins, and nucleic acids [20]. Interestingly, in some cases, fullerenes particles in dark sites may act as antioxidants, avoiding lipid peroxidation induced by hydroxyl and superoxide radicals [53].

The physical interaction between fullerenes and the outer microbial membrane is another antibacterial mechanism, where fullerene NPs induce cell membrane disruption and/or DNA cleavage due to high surface hydrophobicity of the particle, which can easily interact with membrane lipids [30]. As different bacterial species have dissimilar cell wall components, this may account for dissimilar fullerene–cell interactions. Generally, fullerene particles are found to be more biologically-active toward gram positive bacterial species rather than gram negative microorganisms, suggesting that the bactericidal success is reliant on the fullerene insertion into the bacterial cell wall [54]. Experimental data have shown that *P. putida* diminished its levels of unsaturated fatty acids and increased the proportions of cyclopropane fatty acids due to fullerene exposure. This suggests that deterioration of the microorganism related to cell wall damage, namely alterations in membrane lipid structure and membrane permeability, may be an important aspect of fullerene bioactivity [55]. 

From physical point of view, electrostatic forces between fullerenes and a bacterial surface play an important role during their interactions. In order to study electrostatic relations of fullerenes, effects of four forms of fullerene compounds (C_60_, C_60_−OH, C_60_−COOH and C_60_−NH_2_) on *E. coli* and *S. oneidensis* were investigated. The positively charged C_60_−NH_2_, at concentrations as low as 10 mg/L, had an acute effect on cellular integrity as seen in Figure 2B,C, and reduced substrate uptake for both microorganisms [56]. Neutrally charged (C_60_ and C_60_−OH) had mild antibacterial influence on *S. oneidensis*, while the negatively charged C_60_−COOH did not impact the growth of either microorganism. This finding shows that the interaction of positively charged fullerenes with the negatively charged bacterial membranes is more effective than that of neutral and negatively charged fullerene particles [56]. In the same way, several researchers observed the electrostatic attraction to play a major role in the cytotoxic action of fullerene derivatives, causing membrane stress mediated by direct physical contacts, while the role of oxidative stress was considered minor [29]. 

The bacterial respiratory chain (located in the membrane) can be also affected by fullerene particles, signifying one further bacteriostatic mechanism [57]. It is quite possible that fullerene nanoparticles interfere with the cellular energy metabolism chain as opposed to physically disrupting the bacterial membrane. The high concentrations of fullerene derivatives possibly increase the uptake of O_2_, triggering an increase in its conversion to H_2_O_2_, which in turn interferes with the respiratory chain [58]. 

Theoretical approaches (simulations) have also been used to predict the mechanisms by which fullerenes penetrate microbial membranes. Molecular dynamic simulations showed that C_60_ translocation is highly dependent on the specific lipid structures of the target pathogen. C_60_ has a limited tendency to enter homogeneous bilayers of incomplete core lipopolysaccharides, but the translocation of C_60_ into bilayers of complete core lipopolysaccharide is not a thermodynamically favored process. The same simulation revealed that small changes in temperature, ambient ion concentrations, lipopolysaccharide core sugar length, or the incidence of phospholipid defects result in large differences in the interactions between the C_60_ and the surface membranes [59]. It is important to note that the bio-reactivity of nanomaterials toward biological targets not only depends on the cell wall structure but also is subject to cellular enzymes and metabolic activities of the microorganism [60]. Bearing in mind the influence of ambient conditions and microorganism cellular activities will help to understand the inconsistent toxicological results observed in aforementioned fullerene studies. 

Various types of functionalization are being subjugated to fullerene compounds with the aim to control their interactions with biological molecules. The combination of the carboxy-functionalized fullerene into the microbial wall was identified, proposing that the antibacterial performance initiated through its insertion into the cell wall and damaging followed to the membrane’s integrity [61]. In order to study antimicrobial activity of fullerene with different functionalities, two C_70_-derivatives were fabricated [62], namely one with a decacationic side chain (LC17) and another with the same decacationic side chain plus an extra deca-tertiary-amine side chain (LC18) [62]. The decacationically-terminated C_70_ was highly efficient as a broad-spectrum antimicrobial photosensitizer capable of eradicating six logs of both gram-positive and gram-negative microorganisms. Interestingly, the attachment of an additional arm allowed the moiety to act as an effective electron donor and improved the generation yield of hydroxyl radicals under UVA illumination. The white light was more bio-active with LC17, whereas UVA light was more bio-active with LC18 [62]. 

Modification of cationic C_60_ with iodide could create powerful bactericidal fullerene. The antimicrobial mechanism of cationic C_60_/iodide may involve photo-induced electron reduction of ^1^(C_60_>)* or ^3^(C_60_>)* by iodide generating I or I_2_, followed by successive intermolecular electron-transfer actions of (C_60_0>)^−^ to yield reactive radicals [63]. It is worth mentioning the ability of fullerene materials to generate ROS is strongly influenced by chemical modification of the cage [64]. For example, the rate of ROS (singlet-oxygen) production is slower for functionalized C_80_ than for the un-functionalized fullerenes [64]. Often, chemical fictionalization of fullerene particles reduces bond angles from 120° in sp^2^ down to 109.5° in sp^3^, rendering the modified molecule more stable [65].

Fullerenes are highly insoluble in water, however uniformly-distributed aqueous suspension can be prepared using fullerene derivatives [66]. Fullerene suspensions (e.g., aggregations of C_60_) are identified to possess biological activities against microorganisms, possibly distinct from those of bulk solid fullerene [67]. In aquatic systems, it has been argued that the fullerene particles will not necessarily puncture the microbial cell nor generate ROS, but instead exert toxicity as a particle via chemical interactions upon direct contact [68,69]. In this regard, it demonstrated that fullerene aqueous solution is an efficient photo-induced antibacterial agent, even at low concentration (C_60_ = 1.8 × 10^−2^ mM) able to effectively suppress the growth of gram-positive microorganisms [70]. C_70_ suspension is demonstrated to be more photoactive than nC_60_ (forming more ^1^O_2_ than nC_60_ for wavelengths 300–650 nm) resulting in a consistent ratio of 1.69 ± 0.05 times the ^1^O_2_ creation of nC_60_ [71]. Similarly, suspension of C_60_/pyrrolidinium is an extremely active broad-spectrum bactericidal photosensitizer, capable to eradicate more than 99.99% of bacterial and fungal cells in vitro once irradiate with white light [72]. Moreover, the irradiation of dissolved polyhydroxylated fullerene (fullerol) by UV radiation (310 to 400 nm) significantly increased the inactivation of bacteriophage MS2 (up to 4 log) due to ROS generation. In the absence of UV, fullerol revealed limited biological activity due to negligible ROS production [73]. However, generally, one shortcoming of soluble functionalized fullerenes is lying in their absorption range, which is inclined toward the blue and green visible spectrum rather than the red/far red band that have better tissue penetration [74]. Likewise, unmodified fullerenes such as C_60_ have high hydrophobicity and innate tendency to aggregate, preventing efficient photo-activity [45]. It is important to mention that the antibacterial performance of fullerene suspensions is also influenced by the preparation methods used. For example, aqueous fullerene suspensions were prepared using four different procedures, namely using tetrahydrofuran (THF) as a solvent (THF/nC_60_), sonicating C_60_ dissolved in toluene with water (son/nC_60_), stirring C_60_ powder in water (aq/nC_60_), and using a solubilizing agent (PVP/C_60_). All four fullerene derivatives revealed antibacterial activity toward *B. subtilis*, where THF/nC_60_ appeared to have a more effective antimicrobial outcome than the other preparations due to variability in the extraction method [75]. 

To summarize the fullerene antibacterial activities:

- Fullerene is capable of inducing cell membrane disruption and/or DNA cleavage in microorganisms.
- Fullerenes can inactivate microorganisms by impacting their cellular energy metabolism chain.
- Upon light illumination, fullerenes generally yield high rate of ROS that increase the antibacterial performance.

### 3.1. Carbon Nanotubes (CNTs)

In 2007, Kang et al. reported the first article presenting strong antimicrobial performance of single wall carbon nanotubes (SWCNTs) against *E. coli* pathogen [32]. Later, numerous studies proved that both SWCNTs and multi-wall carbon nanotubes (MWCNTs) have powerful inhibitory effects against microorganisms even after short exposure time, suggesting CNTs as an effectively agent for biomedical applications [76,77,78]. From a toxicological point of view, single-walled carbon nanotubes have demonstrated significantly higher antibacterial performance in comparison with MWCNTs (and even fullerene-C_60_) [79]. Despite the incredible material properties and vast number of reports on their antibacterial effects, the bactericidal mechanism of CNTs are yet to be fully understood. 

The mechanism of carbon nanotube toxicity is highly influenced by several factors such as diameter, length, residual catalyst, electronic structure, surface functional group, surface chemistry and coatings of the CNT [80]. In particular, the length of nanotubes is crucially important during interactions with the cell membrane. The shorter tube is founded to exert higher bactericidal performance in comparison to longer tubes [33]. The shorter length may increase the chances for interaction between open ends of nanotubes and a microorganism, leading to extra cell membrane damage [34]. Interestingly, it was observed that when the length of MWCNTs reach up to 50 μm, the tube wrapped around the surface of microbial cell and consequently promoted osmotic lysis of the microorganism [81]. Unlike the solid surface, the interaction of CNTs with cells in a liquid medium is quite different. Longer nanotubes have exhibited higher antibacterial performance than shorter ones. In a liquid system, the short length of CNTs are more likely to self-aggregate without involving a large number of microbial cells, while longer nanotube aggregates are more bio-effective as they affect a larger number of cells in the aggregates [82]. It is well known that aggregation/agglomeration of CNTs is inevitable owing to their unique configuration and powerful van der Waals interactions [83,84].

The diameter of a tube also plays a significant role in the bacterial inactivation process. Smaller diameters can promote damage to cell membrane through the cell-surface interaction [85,86]. Direct microscopic observations have shown that individual CNTs (small diameter ~1.5 nm) attached at one end to the microorganism, protruding from the cell surface with the other end much like ‘needles’ of the hedgehog. Furthermore, the small tubes made the bacteria interact closely with each other. CNTs with large diameter (~15–30 nm) mostly interact with bacteria by their side walls, where the cells were just located on top of the carbon tubes instead of tightly interacting with CNTs bunches [35]. Due to nature of the shape, CNTs maybe have less bactericidal action toward rod-shaped bacteria when compared to spherical ones [81]. 

Another important factor affecting the antimicrobial efficacy of CNTs is emanated from their electronic structure. As known, several parameters (e.g., tube diameter, orientation of the tubes and wrapping angle) highly influence the electronic conductivity of SWCNTs, and small differences in these features could shift the tubes from a metallic to semiconducting state [87,88]. Interestingly, reports have revealed that electronic structure is a significant element governing the antimicrobial action of SWCNTs. The loss of bacterial viability was observed to be positively related to the ratio of metal SWCNTs in samples of similar length, diameter, and number of defect spots [89]. 

CNTs are well known to possess excellent electrical conductors with high dielectric breakdown strength and possess outstanding field electron emission properties [90]. Employing CNTs as a discharge electrode in the corona plasma system may significantly reduce the threshold voltage of plasma field. Hence, the CNT-corona discharge system can produce a low operating voltage and yield a low ozone concentration, which can be used to eradicate bioaerosols. The corona discharge system employing CNTs was reported to inactivate 97% of *E. coli* bioaerosol cells at discharge voltages of −7.5 kV, that is significantly higher than using stainless steel electrodes (the inactivation efficiency is 34% at the same voltages) [91]. The electrode behavior can be expanded further by integrating CNSs with several dielectric polymers such as polydimethylsiloxane (PDMA) and polyvinylidene fluoride (PVDF) fibers, which can be used for several applications (e.g., nanoscale aerosol filters, biomedical implants, artificial muscles and robots, sensors) [92,93].

**Figure 3:**
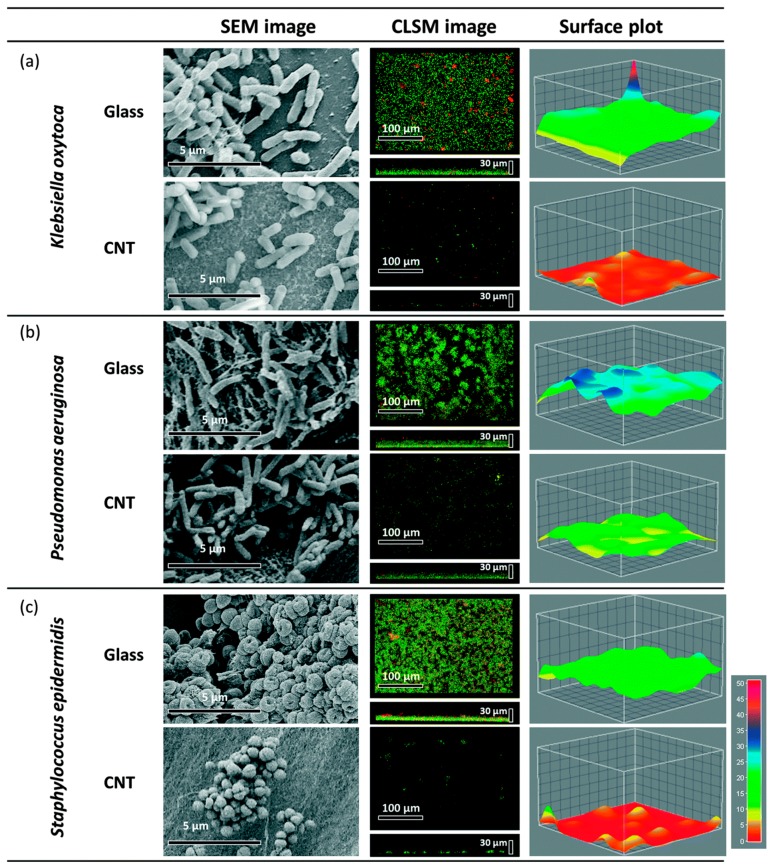
Scanning Electron Microscope (SEM), Confocal Scanning Laser Microscopy (CLSM) and surface plots of biofilm formation of *K. oxytoca* (**a**); *P. aeruginosa* (**b**) and *S. epidermidis* (**c**) on multi-wall carbon nanotubes (MWCNT) (tube length 540 μm) and glass control. Reprinted with permission from Reference [100].

The lethal effects of CNTs on biofilm formation also have been studied to evaluate their potential to impede microorganism attachment and proliferation at different stages of bacterial colonisation. Biofilm structure provides significant protection for bacterial cells and renders them highly resistance to detachment by physical forces and harmful nanoparticles [94,95,96]. CNTs have shown strong bactericidal activities towards cells in biofilms, as seen in Figure 3c–e. It has been reported that microscopic examinations to the bottom layer of the biofilms of *E. coli* and *B. subtilis* in direct contact with coatings containing SWCNT showed that ~80–90% of the microbial cells were dead [97]. Yet, the interaction of CNTs with biofilm is highly dependent on the stage of biofilm formation, where the efficacy of CNTs is more pronounced at the early steps of biofilm formation [98]. As soon as microorganisms become protected within the structure of the mature biofilm, they are less susceptible to the influence of CNTs than bacteria in other biofilm phases [99]. 

The soluble exopolymeric substances that are secreted by microbial cells in the biofilm, at the mature stage, may play the key role in mitigating the lethal effects of carbon tubes [99]. It is worth to mention that the anti-adhesive effect can be caused by the mobility of CNTs, which create an unstable substrate, and thereby affecting appropriate microbial adhesion. Furthermore, the biofilm inhibition is reported to increase with the increasing CNT length, suggesting that longer tubes are more flexible and may oscillate, preventing microbial settlement, as seen in Figure 3 [100]. Also, vertically-aligned arrays of pristine CNTs demonstrated strong repression toward biofilms initiated by *B. subtilis* (biofilm coverage only 6.18% from the substrate), with only individual microcolonies able to form on the surface. These arrays are consisting of tubes much smaller than the usual size of the bacterial cell (~2 μm), which prevents the penetration of microorganisms in between the nanotubes [101]. Interestingly, CNTs are capable to significantly impact biofilms in liquid system. CNTs inhibit microbial biofilms in a concentration dependent mode: 50 μg mL^−1^ SWCNTs reduction the biofilm by 81.19%, and ≥200 μg mL^−1^ SWCNTs totally inhibit the biofilm [98]. However, more in-depth understanding of how CNTs interact with biofilms is needed to engineer appropriate nanomaterial agents to effectively disturb microorganisms at any growth phase or biofilm stage.

In order to evaluate the photo-activities, three carbon nanostructures (fullerene C_60_, SWCNTs and MWCNT) were tested for ROS production under UV irradiation. For similar carbon concentrations, SWCNTs exhibited the highest ROS generation followed by MWCNT, and fullerene [102]. Thus far, several attempts have been made to improve the photo-activity of CNTs through the engagement with various metallic elements. It has been reported that the TiO_2_/MWNTs/Si surface (annealed at 400 °C) displayed great photo-catalyst activities and killed virtually all *E. coli* cells upon contact (in 60 min under the visible light illumination). The Ti–C and Ti–O–C carbonaceous bonds, created at the TiO_2_/CNTs interface, become active upon visible light absorption, and efficiently contributed to the charge transfer between the photo-excited CNTs and the TiO_2_ film, accordingly increasing the rate of generation of OH radicals [103]. Furthermore, branched CNTs were prepared to develop tree-like nanocomposites of TiO_2_/branched-CNTs, which revealed highly enhanced photo-catalytic behaviour against *C. albicans* in comparison with the TiO_2_/CNTs and TiO_2_ thin film. The outstanding visible light-induced biological efficacy of TiO_2_/branched-CNTs is related to the creation of electron—hole pairs by light irradiation with a low recombination rate, as well as the high surface area available for the heterostructure–cell interactions [104]. 

However, in some cases, the presence of metal particles on bioactive CNTs may negatively affect their antibacterial performance. For example, vertically aligned-MWCNTs arrays were deposited in tip-growth method on Ni/Si substrates using PECVD. The results showed that the Ni-removed Ag–CNTs exhibited a powerful bactericidal performance in the dark (inactivation of ~93% in 60 min), whereas a partial antibacterial activity was observed on the films of Ni-removed CNTs and the Ag–Ni/CNTs (inactivation of ~42% and 31% in 60 min). The Ni seeds performed as obstacles preventing active cell membrane rupture during contact between the microorganisms and the tips of the CNT structures [105]. 

It is worth mentioning that collection of CNSs-microscopic data (e.g., SEM and TEM images) requires irradiation of the sample with highly energized electron beams that potentially cause severe catastrophic damages/changes to the structure of CNSs. These damages include heating, electrostatic charging, ionization, displacement damage and sputtering [106]. Crespi et al. reported several high-resolution of CNTs showing anisotropic collapse of the nanotube during microscopy images [107]. Thus, several advanced techniques such as spherical aberration corrected electron microscopy have been developed in order to reduce beam damages in samples, which improved, larger and faster direct-detection electron-counting for images. Also, the aberration correction was found to be very effective to produce two dimensional images to probe the sub-atomic level details of the samples [108].

In many cases, covalent/non-covalent functionalization are conducted to bring functional groups to the surface of nanotubes with the aim to improve their biological performance [109]. For example, sugar with a terminal amino group was used for modification of SWCNTs to control their aqueous solubility and biological activity in binding assays with pathogenic bacteria [110]. Furthermore, treatment of CNT–ZnO with acid groups exhibited stronger photo-inactivation of the bacterial cells than that with the un-functionalized tubes. The functionalized CNT–ZnO inactivated 100% of *E. coli* cells within 10 min of UV-visible light illumination, while the un-functionalized CNT–ZnO could inactivate only 63% of the microorganisms under the same conditions. The higher photo-catalytic action of CNT–ZnO can be attributed to the increases in charge transfer through Zn–O–C carbonaceous bonds created between the Zn atoms and oxygen atoms of the carboxylic groups of the functionalized tube [111]. In this regard, the presence of the amino side group on CNTs increases the positively cationic nature of the structure, which leads to an increase in the efficacy of interactions between the nanotubes and the negatively charged microbial walls [112]. It is important to mention that the functionalization/chemical modification of carbon tubes is fundamentally different for both carbon tube types. In the case of SWNT (a one-atom-thick layer), covalent functionalization will break several carbon double bonds (C=C), leaving vacancies/holes in the nanotube’s configuration and thus varying physical and chemical properties. In the case of MWNT, only the outer wall will be structurally modified [113]. For example, studies showed that SWCNTs with surface functionalities of −OH and −COOH displayed extremely strong bactericidal activity toward both gram-positive and gram-negative bacteria (in DI water and 0.9% NaCl solution), while MWCNTs with similar functionalities did not display antimicrobial action to either type of microbial cells [35].

Introducing small quantities of CNTs into a polymer network can also result in a significant increase in the antibacterial performance of that polymer. Remarkably, polymer with only 3% of SWNTs (0.03 mg/mL of SWNT) demonstrated similar or stronger bactericidal performance than the surfaces consisting of 100% SWNTs [97]. Correspondingly, introducing SWCNTs (diameter 0.8 to 1.2 nm) into a biomedical polymer, e.g., poly (lactic-co-glycolic acid), notably increased the antimicrobial activity of the polymer, with ~98% of bacterial cell dying within one hour of exposure [34]. Similarly, combining SWCNTs with polyvinylpyrrolidone–iodine, a medical polymer, created a porous thin coating, where nanotubes are covered with a monolayer polymer. In these coatings, iodine is attached covalently to the external surface of the porous matrix, promoting it to be gradually released into the system and extend the duration of antibacterial events [114]. 

From the literature, the main antibacterial mechanisms of CNTs can be summarized to include:

- Disruption of membrane integrity by powerful electrostatic forces between microbial outer surface and CNTs, leading to oxidation of the membrane.
- Reactive oxygen species generation may directly harm biological molecules of bacteria and/or indirectly prompt DNA destruction.
- Impurity components (e.g., metallic nanoparticles, catalysts, suspension) that are introduced into CNT-structures during fabrication processes can contribute in their antibacterial activities [80,82,115].
- It is rationally possible to expect that some of antibacterial mechanisms associated with C_60_ can be applicable to SWCNT, in particular the bactericidal oxidative stress, since they are both are made of pure carbon and have similar diameters [116].

### 3.2. Graphene

Graphene-related families including pristine graphene (pG), graphene nanosheets (GNS), graphite (Gt), multilayer graphene (MLG), graphene oxide (GO) and reduced graphene oxide (RGO) have been widely explored. In general, it was found that under similar conditions, GO shows the highest antibacterial activity toward *P. aeruginosa*, followed by rGO, Gt, and GtO [36]. Still, the diverse intrinsic properties of graphene materials (e.g., sheet sizes, layer number, nanopores, shapes, presence of oxygen groups, defect density, quality of the individual graphene sheets, corrugation, hydrophilicity, etc.) make it challenging to predict their exact antimicrobial mechanisms [117]. In order to understand how these nanomaterials interact with microorganisms, several scenarios have been examined. 

**Figure 4:**
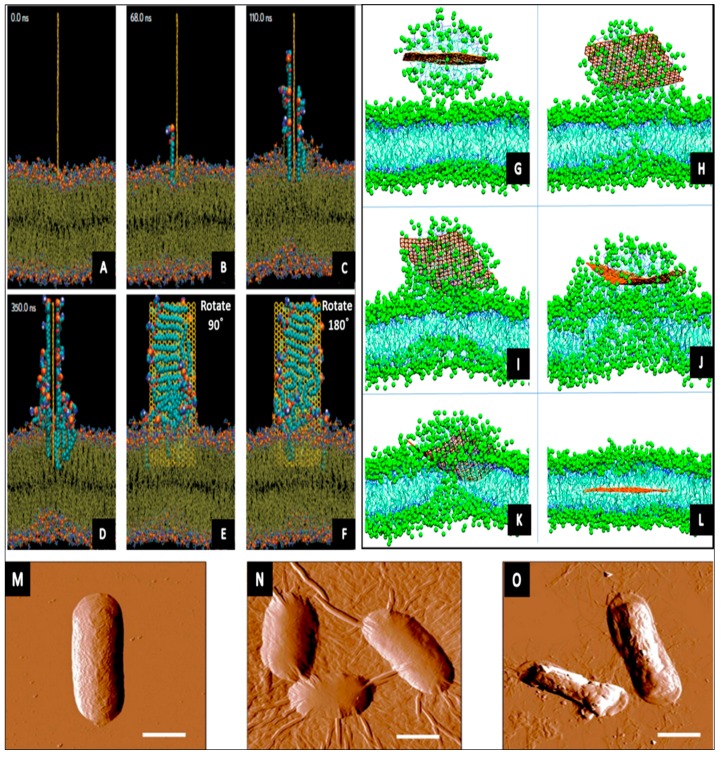
Simulation 1 (**A**–**F**) shows lipid extraction by a graphene nanosheet. An illustrative route of a fully restrained graphene docked at the surface of the outer membrane. The simulation time is shown in each snapshot; e and f are rotated counterclockwise by angle (90° and 180°) from its previous view. Reprinted with permission from Reference [37]; simulation 2 (**G**–**L**) describes the process of self-insertion of graphene sheet into the phospholipid membrane. A graphene sheet merges with the membrane and releases the monolayer that enters the membrane. The snaps are taken at t **G**–**L** = 2.9, 52.4, 120.0, 299.2, 356.4, and 516.4 ns, respectively. Reprinted with permission from Reference [120]; representative AFM images showing *E. coli* cells after incubation with: (**M**) deionized water without GO for 2 h; (**N**) 40 μg/mL GO-0 suspension for 2 h, and (**O**) the 40 μg/mL GO-240 suspension for 2 h. These images reveal the lateral dimension-dependent antibacterial performance of GO nanosheets. Larger GO sheets are covering most of the bacterial cell surface during the interaction compared to smaller nanosheets. The scale bars are 1 μm for all images. Reprinted with permission from Reference [131].

Theoretical simulations and experimental approaches together revealed that physical damage of the microorganism can result from the interaction with graphenes through two possible mechanisms: by severe insertion and cutting of the cell membrane, and by destructive extraction of phospholipids from lipid membranes [118]. The molecular dynamic simulation described that a graphene sheet ‘suspended’ above the bacterial membranes (at a vertical distance of 3.5–4.7 nm) can insert into both outer and inner membranes. The process of insertion begins when the thin graphene sheet starts to vibrate, back and forth, for a period of 10–100 nanoseconds [37]. Then, the atomically thin sharp edged sheet moves and punctures the cell membranes due to the powerful van der Waals interactions with the lipids and hydrophobic effects. The nanosheet intensely extracts the phospholipid molecules from the lipid layers of the membranes. The extraction of phospholipids causes a sparser lipid bilayer and a distortion of the membrane due to powerful dragging forces from the graphene sheet, consequently resulting in irreversible damage to living systems [37,119]. Figure 4 shows simulations of the interactions between graphene nanosheet and bacterial surfaces.

The physical size, in particular the length and surface area, of graphene nanosheets play a substantial role in their antibacterial activity. A simulation showed that a small graphene sheet (~5.9 × 6.2 nm^2^) is likely to slowly diffuse through the phospholipid bilayer membrane [120]. Small nanosheets can enter the membrane without disturbing the order of the phospholipid molecules, while larger sheets (~11 nm) strongly affect the order, the density and the distribution of the phospholipids [121]. On the other hand, interestingly, it has been suggested that direct contact with graphene edges is not an important part of the mechanism. Instead, the availability of the basal planes of GO nanosheets governs whether it is biologically active toward microorganisms or not [122,123]. It is worth mentioning that once graphene sheets are on/within a bacterium, a near-IR light irradiation could kill the cell by immoderate local heating since graphenes can absorb the near-IR irradiation unlike bacteria, which are transparent in this spectroscopic range [124,125]. 

The oxidative stress mechanism has been proposed as a main cytotoxicity mechanism of graphene [126]. The oxidative stress is often mediated by graphene-based materials through the ROS generation. The over-production of ROS can cause cells to enter a state of oxidative stress that results in extensive damage to cellular components, such as lipids and proteins [60]. The oxidative stress is a key process for bactericidal activity of GO through superoxide anion formation, which leads to the permanent DNA laddering that potentially can kill the cell [36]. Importantly, defect densities on graphene sheet surfaces could also contribute to oxidative stress-related antibacterial events by increasing oxygen adsorption at the defect locations [127]. In this regard, the AFM-based force spectroscopy technique was utilized to point out that GO/bacteria interactions are predominantly repulsive due to lipopolysaccharide bridging, clearly emphasizing the role of oxidative pathways in graphene antibacterial mechanisms [128].

Nevertheless, additional reports have highlighted that oxidative stress can be triggered without ROS generation, by means of graphene interference with a specific bacterial process through oxidization and disruption of vital biological structures [129]. It was found that reduced graphene nanowalls (RGNWs) revealed higher antibacterial activity compared to unreduced graphene oxide nanowalls (GONWs). The antibacterial action was explained by the presence of sharper edges in RGNWs, which serve as good electron acceptors and promote more powerful interactions with cell membranes and/or enhanced charge transfer between the bacterial surface and the reduced nanowalls [130]. In order to shed more light on the role of electron transfer phenomenon, three types of monolayer graphene on different substrates (conductor (Cu), semiconductor (Ge) and insulator (SiO_2_)) were investigated against both gram-negative and gram-positive bacteria. The graphene layers on Cu and Ge can remarkably impede the growth of both bacteria, while graphene on SiO_2_ did not show significant influence toward both species. The powerful antibacterial performance of graphene on Cu and Ge can be attributed to the electron transfer. In this case, graphene/substrate junction performs as an electron pump, where electrons are progressively pumped away from the microbial membrane. The electron transfer can cause a ROS-independent oxidative stress in the affected microorganisms [21]. 

Due to its unique dimensional properties, the aggregated graphene sheets in suspensions can trap and isolate bacterial cells from their microenvironment. This physical disconnection from their immediate surrounding will prevent vital glucose consumption and lead to inactivation of bacteria, reducing its ability to proliferate [124]. It has been reported that the antibacterial action of GO sheets against *E. coli* is lateral size dependent, by way of larger nanosheet displaying higher bactericidal performance than smaller ones. Large nanosheets more easily cover the bacterial cells, where cells cannot grow once are fully covered. On the other hand, small sheets may attach to the microbial surface, but cannot fully isolate the cells from their microenvironment, as seen in Figure 4 (N and O) [131]. 

As known, chemical components with various functionalities, e.g., hydroxyl, carboxyl, amide, phosphate, carbohydrate, etc. form the structure of bacterial walls, making the surface of bacteria negatively charged [132,133]. On the other hand, several graphene materials, such as GO, are rich in oxygen-containing groups that are also negatively charged; thus, the surfaces of GO and bacteria would repel each other upon interaction. Often, hydrogen bonds form between GO sheets and the lipopolysaccharides, enhancing the attachment of the nanosheets to the bacteria [134]. However, presence of positively charged nanoparticles on the surface of graphene materials will substantially decrease the negative charge of the resultant nanocomposites (nanoparticles/GO). Due to its positive charge and broad-spectrum bactericidal activity toward numerous pathogenic bacteria, silver nanoparticles are preferred for combination with graphene materials. The Ag/graphene nanocomposite has potential to reduce the surface charge, increase the photo-activity, and synergistic antibacterial properties of individual graphene nanosheets. The GO/Ag was reported to have significant antibacterial activity when compared to individual GO and Ag [134]. The composites of rGO–Au were also confirmed to have a potent bactericidal performance, at ~100% killing efficacy toward both gram-positive and gram-negative microorganisms [135]. More recently, large-area graphene monolayer wrapped silver nanowires were syntheses using CVD. Remarkably, the graphene coverage did not decrease the antimicrobial influences of underlying AgNWs. Quite the opposite, the graphene treatment provided considerable advantages, maintaining robust antibacterial performance under tough environmental conditions and showing up to 100% microbial reduction through the electrolysis of water [38]. Similarly, a sandwich-like nanomaterial based on Ag/halloysite nanotubes/rGO showed greater bacteriostasis facility (~100% against both *E. coli* and *S. aureus*) compared with individual Ag nanoparticles, rGO nanosheets or their composites. This phenomenon may relate to the synergistic antimicrobial activity of Ag and rGO [136]. Nevertheless, introducing Ag NPs into graphenes may increase the antibacterial activity of the nanoparticles. For example, the combination of Ag and rGO in a hybrid nanomaterial deliver a negatively charged surface for Ag particles, leading to minimal agglomeration of the Ag nanoparticles. The graphene nanosheets served as a delivery system, adhering to the bacteria and increasing the contact between the Ag and the microbial surface [137]. Furthermore, introducing nanoparticles such as iron oxide into graphene-related materials rendered them superparamagnetic, which is a valuable attribute for drug delivery systems [138].

The large surface area and the incidence of active groups (e.g., hydroxyl and carboxylic) of GO nanosheets have encouraged researchers to explore its surface functionalization procedures. In a recent report, GO samples were synthesized with different levels of oxidation, hydroxyl, and carbon radical (^•^C). The GO with the highest level of ^•^C exhibited the most powerful bactericidal properties through membrane binding and lipid peroxidation. The bactericidal mechanism of GO/^•^C can be explained by a three-step process: (i) electron transfer from the ^•^C to one of the C atoms adjacent to C=C in the lipid; (ii) subsequently electron transfer from this particular C atom to adjoining molecular dioxygen, creating a lipid peroxide radical involving the attached –O–O bond; and (iii) formation of a lipid peroxide from the lipid peroxide radical [139]. Furthermore, GO nanosheets were modified with hyperbranched polyethylenimine, showing good antifouling and antibacterial effects. Treatment with this hybrid material resulted in a significant reduction in the number of microcolonies of *E. coli* (up to 75%), loss of cellular integrity of bacterial cell membranes and release of cytoplasm [140]. 

Several studies have been carried out in order to introduce graphene-related materials into polymer films. Interestingly, only 3 wt % of GO in a poly(*N*-vinylcarbazole) polymer was able to escalate bactericidal properties up to 30% when used against planktonic cells, and 57% when applied to biofilms, higher than that for untreated GO [141]. More recently, grafting of graphene oxide onto commercial polyamide membranes has been done to promote their antifouling and anti-adhesion properties. The modified membrane revealed a 17-fold decrease in biofouling of *E. coli* (within 24 h) compared to the unmodified counterpart [142]. Likewise, methanol-derived graphene nanosheets loaded with gentamicin revealed a diffusion dominated release mechanism that caused loss of viability in bacteria [143].

The majority of published studies in the literature have established considerable antimicrobial activities of graphene-related materials. Gram-negative bacteria have been shown to be more resistant to the cell membrane damage caused by graphene sheets than gram-positive bacteria, which is potentially related to the existence of the outer membrane layer in the structure of gram-negative organisms [130]. Yet, some bacterial species were found to live in the presence of graphene, such as the *Shewanella* family, which is capable to reduce GO into graphene under ambient conditions with no inhibition of bacterial growth [144]. In some cases, *E. coli* bacteria attached to GO films were able to grow faster and develop denser biofilms than cultures without graphenes, suggesting that GO not only lacks bactericidal activities, but that it basically enhanced bacterial proliferation [145]. In the context of these contradictory results regarding the antibacterial efficacy of graphene-based materials, it is difficult to compare the current data. In fact, new systematic experiments are required to estimate the antimicrobial activities of graphene-related families. 

In summary, the antibacterial mechanisms of graphene-related materials are:

- Serve cutting/damaging to the cell membrane.
- Destructive extraction of phospholipids from lipid membranes.
- Oxidative stress through ROS generation.
- Oxidative stress independent-ROS generation by charge transfer phenomena.
- Separating microorganisms from their microenvironment.

### 3.3. Diamond-Like Carbon (DLC)

Diamond-like carbon structures have been explored extensively for their role as excellent protective coatings in bio-applications. DLC films reveal antibiofouling and antibacterial activities towards microorganisms such as *S. epidermidis*, *S. aureus* and *P. aeruginosa* in vitro [39,146]. The bacterial adhesion on the DLC is relatively related to their sp^2^ and sp^3^ hybridization, and by decreasing sp^3^/sp^2^ ratio, the antibacterial performance is noticeably enhanced [147]. Usually, DLC comprising high fraction (>80%) of sp^3^ bonds is preferred for biomaterial coatings due to its good interaction with human cells and greater wear and corrosion resistance [148]. In order to interpret the bactericidal performance of DLC, several mechanisms have been proposed. One mechanism could be related to the direct physical damages to microorganisms during contact with pure DLC, causing intense membrane impairment and a release of microbial intracellular metabolites [149]. Other researchers suggested that the antimicrobial activity of DLC comes from their chemical inertness due to weakening of the chemical interface in bacterial adhesion process [150]. In many cases, the mechanism of DLC films can be varied based on the microbial species. For example, DLC and DLC/germanium-doped coatings exhibited significant antibiofouling effect against gram-negative bacteria (~90% reduction in biomass), yet did not significantly inhibit gram-positive bacteria [40]. It is worth to mention that there is contrary evidence that show that DLC surfaces have very weak or non-existent antibacterial activities [151,152,153]. 

The bactericidal efficacy of DLC films is critically associated with their surface profile, including high smoothness, high dispersive component of the surface energy and hydrophobicity [154]. In particular, strong hydrophobicity of DLC may cause variations of the bacterial cell membrane and lead to the biological death [155]. Further, the surface free energy is a significant factor controlling DLC antibacterial performance. Often, the surface energy value of DLC films is carefully chosen for specific applications. In order to change the value of surface energy, several elements can be introduced to DLC films. For example, addition of fluorine groups produces bonding modifications by decrease of C–CF bonds and increasing CF and CF_2_ bonds in DLC, enhancing the films’ antibacterial efficacy due to the increase of the work of adhesion of the films for bacteria [156]. The incidence of fluorine can change the wettability of DLC by decreasing the surface free energy and increasing the contact angle [157]. It is well-known that the initial attachment of microorganisms (e.g., *P. fluorescens*) is highly associated with the total surface energy, as the number of adhered cells is reduced with decreasing the total surface energy of the films [41]. Thus, considering surface parameters of DLC will be helpful to design bactericidal coatings, through optimisation of the surface energy.

Inorganic nanoparticles are often incorporated in order to trigger/enhance antibacterial properties of DLC. In theory, introducing a metal particle can acts as a catalyst for the foundation of sp^2^-rich boundary sites in DLC structure [158,159]. It has been observed that low concentration of Ag may reduce the amount of carbon atoms bonded in sp^2^ configuration that promote sp^3^ bonding, whereas at a higher contribution of silver content, the sp^2^/sp^3^ ratio increases [160]. Similarly, copper nanoparticles are well known to enhance the bactericidal activity of DLC. Experimental data indicated that the antimicrobial activity of a-C:H can be significantly increased up to 99.9% once the copper is incorporated (larger than 58.76 wt %) [158]. Further, copper has potential to change the wetting properties of the DLC, which importantly influences the degree of bacterial adhesion. For example, pure DLC surfaces have water contact angle of around 66.8°, but when the Cu concentration increased from 0.1 to 7.0 at %, the contact angle increased from 76.6 to 82.7°. Once the Cu concentration reached 24.4 at %, the contact angle enlarged significantly up to 104.4° [161]. DLC films with hydrophobic properties may increase the bactericidal performance [162]. In some cases, metallic nanoparticles maybe have drawbacks to the DLC matrix. For example, adding silver increased the hydrophobic and antimicrobial outcomes of a-C:H materials, but is accompanied with the shortcoming of lower hardness. Further increases in silver content did not positively contribute to the enhancement of antimicrobial efficacy, yet caused considerable reduction of surface hardness and flatness [163]. It is not yet understood whether the mechanism of NPs embedded within DLC materials is similar to that of free particles, or whether these particles are acting in a different way [40]. Still, DLC/composite films are engaged successfully as engineered antibacterial coatings due to their ability to govern the release of antibacterial nanoparticles [164].

The surface chemistry of DLC coatings can be controlled by integrating selective dopants (e.g., F, N, Si, B) that enhance a specific property (e.g., bioactivity, corrosion) [165,166,167]. Remarkably, dopants give a possibility to manufacture coatings that have high antibacterial activity and favorable interaction with human cells. DLC coatings contain both Ag and Si (1.65 at % Si and 2.09 at % Ag) are biocompatible and capable to reduce the viability of the adhered bacteria up to 50%, while the non-doped DLC films have no bactericidal effect [168]. Similarly, the existence of nitrogen in DLC films may enhance the antibacterial performance. It was found that higher nitrogen content in DLC coatings is associated with lower numbers of bacterial cells attached to the surface [146]. Boron element also notably changes surface characteristics of DLC. Boron creates B-rich carbide bonds in DLC films that increase the sp^3^/sp^2^ ratio and decrease surface roughness parameter [169]. The high sp^3^ level and low surface roughness typically improve the antibacterial performance of DLC. On the other hand, some dopants may change the surface chemistry and reduce the bactericidal performance of DLC. For example, high content of silicon can minimize the antibacterial efficacy of the films [146]. Above mentioned studies clearly indicate that dopants have major influence on the surface chemistry and bioactivity of DLC.

In recent years, there has been an increasing interest in the use of environmentally friendly approaches to develop DLC. In particular, plasma enhanced chemical vapor deposition (PECVD) is preferred for DLC production as it yields uniform structures with reasonable fabrication rates and appropriate for large-scale manufacturing [170]. In addition, it is easy to control deposition parameters and relatively inexpensive [171]. Recently, PECVD reactor engaged with DC pulsed source (containing an apparatus for liquid delivery) was employed to fabricate DLC and camphor: DLC on medical instruments (polyurethane (PU)). The camphor:DLC/PU and DLC/PU films achieved a reduction of 99% and 91% in the proliferation of *C. albicans* biofilms, respectively [172]. Further, PECVD systems were used to produce DLC and Ag-DLC films that showed bactericidal efficacy by eradicating (in 3 h only) approximately 33% and 68% of the total microorganisms, respectively [149]. It is important to mention that among all deposition parameters in PECVD, the input power is a key factor for controlling the structure and properties of the resultant films. For example, fluorinated-DLC films were fabricated at 200 W of RF plasma (in direct mode) and MW (in remote mode) eradicated 70% of *E. coli* cells. But, increasing MW power up to 450 W led to 30% of bacterial reduction only, which is possibly associated with the decrease of surface roughness and F content [173].

The antibacterial activities of DLC can be summarized:

- Strong hydrophobicity of DLC may cause variations of the bacterial cell membrane.
- DLC films reveal antibiofouling/antibacterial based on their surface profile.
- There is almost a specific property for each DLC film, depending on the fabrication conditions.
- Sp^3^/sp^2^ ratio play often important role in DLC biological activities.

## 4. Carbon Nanostructures Production Challenges

CNSs exist as either individual nanoscale objects or assembled in microscale structures with different degrees of organization, from random to highly-ordered and hierarchic. Although numerous approaches have been developed for fabricating CNSs, efforts pertaining to the large-scale production of CNSs still face serious issues such as low fabrication rate, poor yield (e.g., low carbon consumption and rapid catalyst losses), high variations in material properties [174], and high cost of production (e.g., 50 g of fullerene C_60_ is commercially available for $1245.00) [175]. Further challenges arise when CNSs are employed, particularly, in biological applications. For example, surface-immobilized graphene may effectively and/or selectively restrict colonization of pathogens, but it is slow and expensive to produce, and large-scale fabrication is still very difficult due to the requirement for vacuum chamber. Thus, while it may be appropriate for high-value applications, such as preventing the implant-associated infections, at this stage, it is unlikely to be used in high-volume applications, such as coating of hospital surfaces. On the other hand, CNTs produced in gas phase can then be deposited over large surfaces by, e.g., solution processing, however, then you may lose some biological activity that arises form CNTs being organized in a particular manner, or having the freedom of movement. Unless effectively overcome, these challenges will significantly curb progress of CNSs-based manufacturing and applications.

One more aspect to be addressed is the cost of the precursors that used to synthesis CNSs (e.g., natural graphite, purified hydrocarbon gases and some organic compounds). Typically hydrocarbon gases such as methane, acetylene, xylene, toluene benzene etc. gained increased demand due to the popularity of chemical vapor deposition in producing CNSs. Nevertheless, hydrocarbon gases are refined from petroleum and hence are expensive sources and explosive in nature. In addition, the dissociation of hydrocarbon gases produces mixtures of volatile organic compounds and polycyclic aromatic hydrocarbons as by-products which are harmful and contribute to the greenhouse gas emission [176]. On the other hand, natural resources have the advantage of abundance, low cost and environmental friendliness representing promising precursors for CNS synthesis. To date resources such as biomass, oils, proteins etc. have been tested to develop carbon nanostructures and succeeded. For example, Sun et al. reported the preparation of porous grapheme-like nanosheet from renewable biomass coconut shell [177]. Similarly, Ruiz et al. published the production of graphene from sucrose and gelatine protein through thermal treatment at N_2_ atmosphere [178]. Kawale et al. [179] reported the synthesis and electrical characterizations of carbon nanotubes through hot wire chemical vapor deposition using a wide range of natural precursors such as camphor, mustard oil, castor oil, coconut oil, turpentine oil and menthol on quartz substrates. Noteworthy is that, carbon nanostructures derived from natural precursors have excellent yield and comparable properties with that of the hydrocarbon-based nanostructures.

## 5. Conclusions

Current progress in nanotechnology opens unprecedented opportunities for the advancement of biomaterials. In particular, carbon-based nanomaterials including fullerene, CNTs, graphene and DLC have revealed attractive bactericidal properties that can be tailored to produce innovative nanocomposite materials. The possible mechanisms of their antibacterial action were proposed to be either physical (cell wall damage and cytoplasm separation) and/or chemical effects (oxidative stress and ROS generation). Further, synergetic effects of antibacterial CNSs can be observed in many cases.

It is important to conclude that the biological performance of carbon nanomaterials is highly dependent on various parameters including size, shape, light-presence, functionalities, defect density, electronic configuration, temperature, and nature of the target microorganisms etc. In general, toxicity mechanisms of fullerenes give the impression to be critically dependent on photo-illumination. On the other hand, bactericidal mechanisms of CNTs are greatly related to their dimensional factors such as diameter and length of the tube. Biological activity of graphene appears to be critically relying on the physical size and number of nanosheets layers. DLC bactericidal performance is chiefly connected with their surface profile parameters such as surface energy and hydrophobicity.

Overall, carbon-based nanostructures are promising antibacterial candidates for a wide range of medical applications due to their abilities to kill microorganisms, prevent bacterial adhesion and biofilm formation. Yet, it is not fully understood how these nanostructures can deactivate microorganisms. Further investigations, theoretical and experimental studies are indeed required to clearly elucidate their exact mechanisms.

## 6. Future Outlook

Even though CNSs emerged merely three decades ago, considerable progress has been achieved within this short period of time, and they still fascinate researchers with their impressive bactericidal properties. At the moment, most antibacterial carbon-nanomaterials are still under research/development. Despite the fact that several carbon-allotrope-products are commercially available now, CNSs are incapable to substitute/compete with the currently used antibacterial materials (e.g., polymers, Ag-NPs) for many reasons; for instance their toxicity profile for human cells has not been well-addressed yet, they are slow and expensive to produce, and large-scale fabrication is still very challenging. Thus, upcoming experiments should mainly concentrate on producing non-toxic CNSs [180] in large quantities at minimal production cost. At this stage, it seems that functionalization of CNSs is a promising way to expand their performance in the biological field opening the way for wide-integration in biomaterials.

## Figures and Tables

**Table 1:** The antimicrobial performance of several forms of Carbon nanostructures (CNSs).

CNSs	Type	Synthesis Method	Modification/Catalyst	Dimensions	Concentration	Species of Bacteria	Antibacterial Efficacy	Antibacterial Mechanism	Ref.
Fullerene	C_60_	Four-step reaction	Cyclen-functionalized fullerene derivative	150 to 320 nm	7.5 μg/mL	*E. coli*, *S. aureus*	86.1% 40.7%	Electrostatic attraction plays a major role	[29]
	C_60_	-	Light (160 J/cm^2^ of 385–780 nm)	-	100 μM	*S. aureus*, *P. aeruginosa*, *E. coli*, *C. albicans*	6 log_10_ 1 log_10_ 3 log_10_ 3 log_10_	Increase in membrane permeability	[30]
	C_70_	SES Research production	Ag-NP and polystyrene-(PS-P4VP)	-	2 wt % of PS-P4VP	*E. coli*	~5 log	C_70_ and Ag-NPs, synergistically target bacterial cells that increase photo-generated ROS	[31]
CNT	SWCNT	CO disprop-ortionation	-	d = ~1 nm l = 1–3 µm	5 μg/mL	*E. coli*	86.8%	Irrecoverable damages to the outer membrane, releasing the intracellular content	[32]
	SWCNT	CO incorporated MCM-41	-	d = 0.9 nm l = 2 µm	5 µg/mL	*E. coli*	80.1%	Cells lost their cellular integrity	[33]
	MWCNT	CVD method	-	d = 30 nm l = 70 µm	5 µg/mL	*E. coli*	24.4%	The majority of cells still intact and maintain their outer membrane	[33]
	SWCNT	CO decomposition	Poly(lactic-*co*-glycolic acid)	d = ~1 nm l = 300 nm	1/70 CNT/polymer	*S. epidermidis*	98%	Cells loss their viability and deactivated	[34]
	SWCNT	NanoLab productions	Functional groups (−OH, −COOH, and −NH_2_)	d = ~1.5 nm l = 10 µm	200–250 μg/mL	*S.typhimuriu*, *B. subtilis*, *S. aureus*	~7 log	Form aggregates that act like needles surrounding the cells	[35]
	MWCNT	NanoLab productions	Functional groups (−OH, −COOH, and −NH_2_)	d = 15–30 nm l = 1–5 μm	500 μg/mL	*S.typhimuriu*, *B. subtilis*, *S. aureus*	Minor	-	[35]
Graphene	Graphene oxide	Hummers and Offeman	-	0.525 μm		*P.aeruginosa*	92%	Oxidative stress, ROS generation, and laddering of DNA	[36]
	Reduce graphene oxide	Synthesized from GO	-	3.40 μm	0.1 mg/mL	*P.aeruginosa*	90%	Oxidative stress and ROS generation	[36]
	Graphene oxide	Modified Hummers’ procedure	-	205 nm	100 mg/mL	*E. coli*	-	Extraction of phospholipids from the cell membrane	[37]
	Graphene	low-pressure CVD	AgNW/water electrolysis	-	-	C. albicans	100%	Graphene layer reduces the attachment of microbes	[38]
DLC *	-	DC sputtering	Polytetrafluoroethylene hybrid	t = 200 nm	-	*S. epidermidis*, *S. aureus*	56% 51%	Reduce biofilm formation and cell attachments	[39]
	Multilayer films	Pulsed-DC-PECVD	Germanium	t = 1–2 μm	28.9% germanium	*P. aeruginosa*	62.6%	Disruption to the outer cell wall and leakage of cellular components.	[40]
	Multilayer films	Pulsed-DC-PECVD	Germanium	t = 1–2 μm	28.9% germanium	*S. aureus*	-	Minor reduction in biofilm	[40]
	Two layers (a-SiC:H/F-DLC)	RF-PECVD	Fluorine	t = 1 µm	6.5–39.2 at % F	*P. fluorescens*	48.8%	Reduce bacterial attachment and proliferation.	[41]

* Diamond has carbon atoms in sp^3^ hybridization arranged in a face-centered cubic crystal, while graphite has sp^2^ hybridization structured in a hexagonal close-packed crystal. DLC is a combination of sp^2^ and sp^3^, and the differences in the diamond/graphite arrangements and their chemical bonds cause high variation in DLC properties, showing almost a specific property for each DLC film [42]. Thus, the given values in the above table can be highly varied with fabrication method/conditions.
